# Dengue Virus Pathogenesis: Innate Immune Signaling and Cell Death Receptor Pathways

**DOI:** 10.1002/iid3.70595

**Published:** 2026-11-05

**Authors:** Farjana Islam

**Affiliations:** ^1^ Department of Biochemistry and Molecular Biology Shahjalal University of Science and Technology Sylhet Bangladesh

**Keywords:** cell death receptors, dengue virus, immune modulation, innate immune signaling pathways

## Abstract

**Background:**

The infection caused by the dengue virus (DENV) involves a complicated interaction between viral replication and the body's immune responses. As the first line of defense, the innate immune response limits viral replication but can also lead to immunopathology if it is improperly regulated. The DENV activates several pattern‐recognition receptor (PRR) pathways, such as the RIG‐I‐like receptor (RLR), the Toll‐like receptor (TLR), and the cGAS–STING signaling pathway, all of which control interferon and inflammatory responses.

**Objectives:**

The purpose of this review is to outline the molecular mechanisms involved in the process of innate immune recognition and signaling when DENV infects cells, with a specific focus on the virus's immune‐evasion tactics and the relationship between innate immune signaling and programmed cell death; it also examines the possible implications of these pathways for the development of a dengue vaccine and for antiviral approaches.

**Discussion:**

The RNA of DENV is mainly detected by RNA‐sensing pathways such as the RLRs and TLRs, while cGAS–STING signaling can be triggered indirectly as a result of virus‐associated cellular stress and the presence of cytosolic DNA. DENV counteracts these antiviral responses by acting on key signaling molecules such as MAVS, STING, TBK1, IRF3, and STAT2, thus promoting viral persistence and replication. At the same time, death‐receptor pathways involving tumor necrosis factor receptor (TNFR), Fas (CD95), and TNF‐related apoptosis‐inducing ligand (TRAIL) receptors control caspase‐8‐dependent apoptosis and may play a role in the elimination of infected cells as well as in causing tissue injury and immunopathology. The interaction between innate immune signaling, inflammatory responses, and caspase‐8‐mediated cell death therefore serves as an important factor in determining the balance between antiviral protection and disease progression. If these mechanisms are understood, this could form the basis for developing strategies that boost antiviral immunity while reducing excessive inflammation and pathological cell death.

**Conclusion:**

The way in which dengue disease develops is due to the continuous interaction between the body's antiviral innate immune responses, the virus's ability to evade the immune system, and programmed cell death. It might be possible to enhance control of dengue disease by targeting these closely related pathways, whether by means of vaccine development, antiviral drugs, or therapies that act on the host. Yet any intervention focused on a specific pathway will need to take into account the stage of the disease, the cellular environment, and the possible risk of weakening the protective antiviral immunity.

## 1 Introduction

Dengue virus (DENV) is the most prominent mosquito‐borne RNA viral disease affecting humans visceral and central nervous system, with infections estimated to occur in hundreds of millions of cases annually worldwide [1]. Transmission of DENV is primarily by two vectors such as *Aedes aegypti* and *Aedes albopictus* mosquitoes, which are dominant in tropical and subtropical countries. The virus belongs to the genus *Flavivirus* under the family Flaviviridae, constituting four distinct serotypes (DENV‐1–DENV‐4), representing differences amongst serotypes measuring 25%–30% at the amino acid level [2]. It has been estimated that approximately 3.6 billion people are infected with the DENV annually in more than 100 countries [3]. Clinical outcomes such as asymptomatic infection and classical dengue fever, to severe dengue hemorrhagic fever and dengue shock syndrome, are exposed, followed by infection with any serotype. with significant morbidity and mortality [4].

The pathogenesis of dengue disease is complicated and multifaceted, influenced by viral, host, and immunological factors [5]. Although viral replication is crucial for disease progression, the determination of disease severity is essentially dependent upon host immune responses [5]. The infected mosquito with the virus activates the DENV infection in the host through the injection of the virus particle into the skin [6]. Virus dissemination occurs through the lymphatic system, subsequently infecting the whole bloodstream, where it predominantly infects the spleen and liver [7]. In particular, dysregulation in innate immune responses and excessive inflammation participate in the vascular leakage, hemorrhage, and shock syndrome occurs in severe dengue [8]. Among the host immune signaling pathways during infection, the nuclear factor‐κB (NF‐κB) pathway has increasingly been distinguished as a pivotal regulator of host immune responses in DENV infection [9].

Innate immunity serves as the first line of defense against viral infection by means of pattern‐recognition receptors (PRRs), which detect pathogen‐associated molecular patterns (PAMPs) and trigger signaling pathways that lead to the production of type I interferons (IFNs) and pro‐inflammatory cytokines [10]. The main families of PRRs that play a role in antiviral defense are Toll‐like receptors (TLRs), RIG‐I‐like receptors (RLRs), and the cytosolic DNA‐sensing pathways, such as the cyclic GMP–AMP synthase (cGAS)–stimulator of interferon genes (STING) pathway [11]. Endosomal TLRs, for example TLR3 and TLR7, are particularly capable of detecting viral double‐stranded and single‐stranded RNA, thus causing the activation of NF‐κB and interferon regulatory factors (IRFs) [12]. Furthermore, retinoic acid‐inducible gene I (RIG‐I) and melanoma differentiation‐associated protein 5 (MDA5) respectively detect particular RNA structures by means of activating intracellular adapter proteins such as mitochondrial antiviral signaling protein (MAVS) and tumor necrosis factor receptor‐associated factors (TRAFs), these in turn activating NF‐κB and interferon regulatory factor 3 (IRF3)/interferon regulatory factor 7 (IRF7) immune signaling [13]. NF‐κB is a family of transcription factors that controls the expression of genes involved in immune responses, cytokine production, inflammation, cell survival, and the regulation of cell death. Its activation results in the release of pro‐inflammatory cytokines and chemokines that help the host in resisting invading pathogens [14, 15]. Yet DENV can interfere with antiviral immunity via viral proteins, such as NS2B/3, NS4A, NS4B, and NS5, all of which act on different parts of the innate immune and interferon signaling pathways, including PRR‐associated signaling and the downstream JAK–STAT responses [16]. At the same time, DENV infection can cause NF‐κB‐related inflammatory responses, leading to the production of mediators such as TNF‐α, interleukin‐6 (IL‐6), and interleukin‐8 (IL‐8), as well as inducible nitric oxide synthase [16]. The interaction between impaired antiviral signaling and dysregulated inflammation may be responsible for viral persistence, vascular dysfunction, and tissue injury in severe dengue.

Besides innate immune signals, cell death receptor‐mediated apoptosis reveals a pivotal role in dengue pathogenesis. Death receptors, including TNFR1, Fas, and TRAIL receptors, are stimulated upon ligation of their specific ligands to induce apoptosis, while dengue infection leads to the release of viral particles [17]. Apoptosis of diseased cells may act as an antiviral defense mechanism for restricting viral amplification; however, excessive activation of apoptosis can turn into severe clinical outcomes. Upon DENV infection, disease severity has been associated with higher expression of TNF‐ α, FasL, and TRAIL levels, revealing the excessive viral replication [18]. Apoptosis in endothelial cells makes a great contribution to the damage of endothelial cells, leading to perturbation in vascular function and plasma leakage, represented as severe dengue outcomes [19]. Furthermore, apoptosis of lymphocytes may downregulate adaptive immune function, participating in disease replication.

Innate immune signaling and death receptor‐induced signaling pathways are greatly interconnected for mediating antiviral defense against DENV. Upon activation, PRRs mediate the expression of inflammatory cytokines and earlier immune protective function at the onset of infection [20]. Additionally, cell survival NF‐κB signaling can downregulate death receptor‐induced apoptosis through the expression of antiapoptotic genes such as BCL‐2, BCL‐XL, and XIAP, generating a balance between elimination of diseased cells and restricting excess inflammation [21]. Therefore, the pathogenesis of dengue is intricately balanced between innate immune signaling and apoptotic pathways, in which dysregulation dampens the immune response to limit viral replication and increases infection severity.

Herein, we focus on recent knowledge regarding the molecular mechanisms of innate immune signaling pathways for revealing antiviral effects against the DENV, as well as how the DENV applies strategies for the manipulation of innate signaling pathways. It also discusses how cell death receptors are involved in the pathogenesis of the DENV, which implies CASPASE‐8‐mediated apoptosis. This review will explore the advancement in the immune response, featuring dengue research and the identification of the critical target of vaccine and therapeutic strategies.

## 2 Strategy for Carrying Out the Literature Search

A narrative review of the literature was carried out using PubMed/MEDLINE and Google Scholar in order to find studies relating to DENV innate immune signaling, viral immune evasion, programmed cell death, vaccine development, and antiviral strategies. Papers published between 2000 and 2026 were taken into account, including mechanistic and therapeutic evidence. The search employed combinations of the following terms: “dengue virus,” “DENV,” “innate immunity,” “RIG‐I,” “MDA5,” “MAVS,” “TLR,” “cGAS‐STING,” “interferon,” “JAK‐STAT,” “NS2B/3,” “NS4A,” “NS4B,” “NS5,” “death receptor,” “TNFR1,” “Fas,” “TRAIL,” “caspase‐8,” “apoptosis,” “necroptosis,” “pyroptosis,” “vaccine,” and “antiviral.” Only those studies that were directly relevant to the mechanistic and translational aspects of the review were included, while duplicate and unrelated publications were excluded.

## 3 Pattern Recognition of Dengue Virus

Innate or nonspecific immunity is the first line of defense mechanism in protecting the host against viral replication and contributes to the generation of adaptive immune responses. It involves signaling networks of proteins and kinases that are involved in recognition, regulation, and preventive viral effects [22] (Figure 1). Innate or nonspecific immune response is initiated by PRRs, which are distinguished by their structure, such as polysaccharides, glycolipids, nucleic acids, and nucleoprotein, as well as expression in different cellular components such as plasma membrane, cytosol, and lysosomes [10]. The innate immune system is critical for defending the host against infection during the critical hours or days following exposure to antigens. PRRs are involved in the critical moment to detect the conserved molecular structures known as PAMPs, particularly viral RNA intermediates [22]. Diverse PRRs are crucial components for detecting viral RNA intermediates generated during viral replication (Table 1).

**Figure 1:**
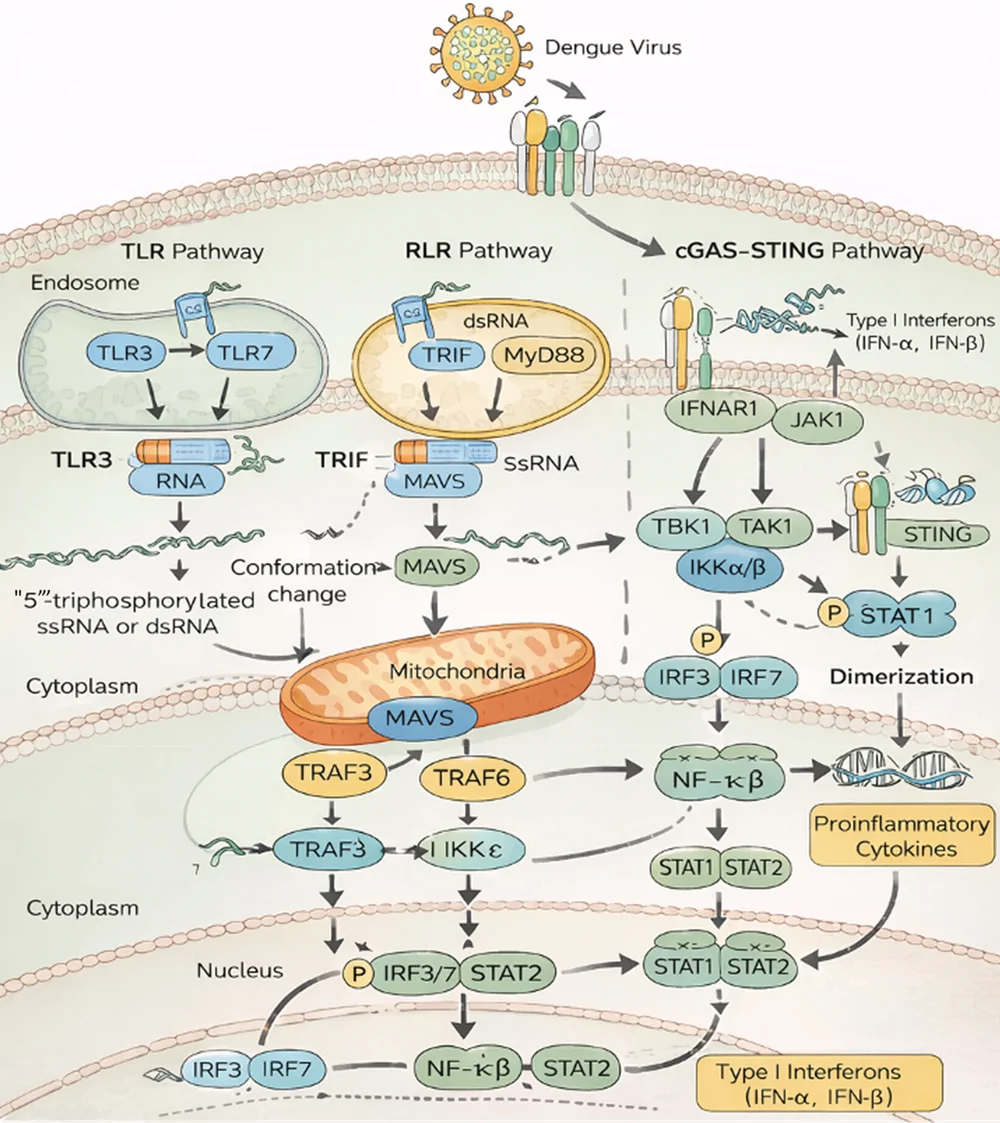
Innate immune signaling pathways activated by dengue virus. Innate immune signaling pathways are activated during dengue virus infection to induce NF‐kB and interferon‐stimulated gene expression for limiting virus amplification and dengue pathogenesis.

**Table 1:** PRRs and DENV‐associated ligands or triggers.

Receptor/pathway	Relevant ligand	Downstream response
RIG‐I	DENV‐associated RNA bearing appropriate recognition features	MAVS–TBK1/IKKε–IRF3/IRF7; type I IFN
MDA5	Long double‐stranded RNA associated with viral replication	MAVS‐dependent antiviral signaling
TLR3	Double‐stranded RNA	TRIF‐dependent IRF3 and NF‐κB signaling
TLR7	Single‐stranded RNA in endosomes	MyD88‐dependent interferon and inflammatory signaling
cGAS–STING	Cytosolic host DNA, potentially including mitochondrial DNA released during cellular stress	TBK1–IRF3 and NF‐κB signaling

*Note:* The major innate immune sensors implicated in DENV recognition and their downstream signaling pathways are summarized in this table.

### 3.1 RIG‐I‐Like Receptor (RLR) Pathway

RLR signaling pathway, presenting RIG‐I and MDA5, is a crucial component of the innate immune response in sensing dengue viral RNA infection [23]. RLRs are present in most cell types and are located in the cytosol, although it has recently been identified in the nucleus. RLR signaling pathways recognize viral replication‐generated RNA and promote the antiviral signaling complex, resulting in interferon and cytokine production [24]. Innate immune sensors RIG‐I and MDA5 are featured by the detection of distinct RNA structures. RIG‐I preferentially detects short double‐stranded or single‐stranded RNA, especially containing 5′‐triphosphate or diphosphate, serving as a short‐type sensor, while MDA5 preferentially detects long double‐stranded RNA, functioning as a long‐type sensor, mediating conformational changes upon RNA binding. In consequence, this conformational change mediates the expression of caspase activation and recruitment domains (CARDs). The exposed CARDs associate with MAVS [25], which is located on the outer mitochondrial membrane.

MAVS functions as a prominent signaling transducer, allowing the recruitment of downstream adapter proteins, such as TRAF. It further results in the recruitment of several kinases such as TANK‐binding kinase 1 (TBK1) and IκB kinase ε (IKKε) [26]. These kinases then induce the phosphorylation of IRFs IRF3 and IRF7, enhancing their dimerization and nuclear translocation. Besides this, MAVS activation also leads to the activation of NF‐κB, which results in the expression of pro‐inflammatory cytokines. The combined activation of IRF3/7 and NF‐κB consequently mediates robust transcription of type I interferons and inflammatory mediators [27]. Upon RLR activation, generated type I interferons function in an autocrine and paracrine manner via interferon receptors, stimulating the JAK–STAT signaling pathway and generating interferon‐stimulated genes, establishing an antiviral state [28]. The RLR pathway is a prominent target of viral immune evasion strategies, establishing its significance in host–virus interactions.

### 3.2 Toll‐Like Receptor (TLR) Signaling

TLR immune signaling contributes as a pivotal innate immune component against DENV infection. TLRs, like other PRRs, are located on the cell surface or endosomal compartments, in which they recognize conserved motifs of PAMPs originating from invading viruses, as well as generated viral RNA intermediates during dengue viral replication, resulting in the induction of antiviral and inflammatory signaling pathways [29]. TLRs such as TLR3 and TLR7 play significant roles in recognizing DENV. TLR3 detects the double‐stranded RNA intermediate of DENV, while TLR7 recognizes single‐stranded RNA, which contains guanosine and uridine residues [30]. These receptors are predominantly expressed in innate immune cells such as dendritic cells, monocytes, and macrophages, which function as critical aspects of DENV infection. Upon ligand binding, TLR3 allows the recruitment of the adapter protein TIR‐domain‐containing adapter inducing interferon‐β (TRIF) for downstream signaling, while TLR7 signals via myeloid differentiation primary response 88 (MyD88) [31]. This allows the further initiation of downstream signaling adapter proteins such as TRAFs and kinases such as TBK1 and IKK complexes. These signaling cascades prompt the phosphorylation and IRF3, IRF7, and NF‐κB, inducing the expression of antiviral cytokines such as type I interferons (IFN‐α/β) and pro‐inflammatory cytokines, including TNF‐α and IL‐6. The type I interferon generated downstream of TLR signaling induces its binding to interferon α/β receptors (IFNAR) 1/2 on virus‐infected cells, mediating the activation of the JAK–STAT pathway, which consequently promotes the antiviral effect through the activation of STAT1 and STAT2 and interferon‐stimulated gene factor 3 (ISGF3) with antiviral effects [27, 32]. However, excessive inflammation following TLR activation has been linked to severe dengue infection, emphasizing the multifaceted role of TLR signals in antiviral effects and immunopathogenesis.

### 3.3 cGAS–STING Signaling Pathway in Antiviral Immunity

The cGAS–STING pathway, which is a key part of the body's innate immune response, is able to detect DNA that is present in the cytosol and then triggers antiviral and inflammatory reactions. When cGAS binds to DNA in the cytosol it gets activated and carries out the synthesis of the second messenger 2′3′‐cyclic GMP–AMP (2′3′‐cGAMP) [33]. This molecule then binds to and activates STING, an adapter protein that is mainly found in the membrane of the endoplasmic reticulum [34, 35]. Once activated, STING brings in TBK1, which in turn causes the phosphorylation of IRF3. The phosphorylated IRF3 forms a dimer and moves into the nucleus, where it causes the expression of type I interferons, especially IFN‐β, as well as other antiviral genes. Furthermore, STING signaling can also lead to NF‐κB‐dependent transcription, thereby contributing to the secretion of pro‐inflammatory cytokines and to the regulation of innate immune responses.

Even though the DENV is an RNA virus, the RNA of its genome does not act directly as a ligand for cGAS. It follows that any role of the cGAS–STING pathway in the host's response to DENV infection must be indirect, rather than involving direct recognition of the viral genome. A suggested mechanism is that mitochondrial dysfunction or cellular stress could cause mitochondrial DNA of host origin to be released into the cytosol, where it might then activate cGAS and lead to downstream STING signaling. Even though this mechanism offers a reasonable connection between cellular damage and the activation of the innate immune response during DENV infection, the extent to which it contributes to antiviral defense may differ depending on the cell type, the infection conditions, and the degree of mitochondrial injury. The evidence currently available therefore has to be treated with caution, especially when applying results from experimental models to human dengue pathogenesis [36, 37].

DENV also uses mechanisms that counteract the host's innate immune signaling. Specifically, the viral NS2B/3 protease complex has been found to cleave human STING, thus providing a means by which DENV could interfere with STING‐dependent antiviral responses [36]. It is important, however, to distinguish this interaction from the direct inhibition of cGAS by the virus, and the degree of impairment of downstream signaling may vary according to the experimental context. More generally, the role that the cGAS–STING pathway might play in controlling DENV is not fully defined. Even though this pathway may shed light on the host's antiviral defense and on possible therapeutic targets, further studies are still required to establish its role in DENV infection and to determine whether modulating it can enhance viral control without worsening inflammatory injury.

## 4 Modulation of Innate Immune Signaling Pathways During Dengue Virus Infection

The DENV has developed a number of mechanisms by which it interferes with innate immune signaling at various stages of the antiviral defense process, such as the detection of viral RNA, the activation of adapters, the production of interferons, and the subsequent JAK–STAT signaling (Table 2). However, the impact of each nonstructural protein is not the same across all DENV serotypes, strains, cell types, or experimental systems. It therefore follows that the current evidence must be understood in a way that is specific to the pathway, rather than being seen as representing a single mechanism common to all DENV isolates (Figure 2).

**Table 2:** DENV proteins and their immune targets.

DENV protein	Host target/pathway	Immune‐evasion mechanism
NS3	RIG‐I‐associated signaling	Interference with RIG‐I translocation through interaction with 14‐3‐3ε
NS4A	RIG‐I–MAVS signaling	Interference with RIG‐I–MAVS association in specific experimental systems
NS2A and NS4B	TBK1–IRF3 signaling	Inhibition of antiviral signaling; effects may vary by serotype
NS2B	cGAS	Promotion of cGAS degradation through an autophagy–lysosomal mechanism
NS2B/3	Human STING	Cleavage of STING, disrupting downstream signaling
NS5	STAT2/JAK–STAT	Binding to STAT2 and promotion of its degradation, reducing ISG expression

*Note:* The major DENV proteins, their host target, and immune‐evasion mechanisms associated with DENV proteins are summarized in this table.

**Figure 2:**
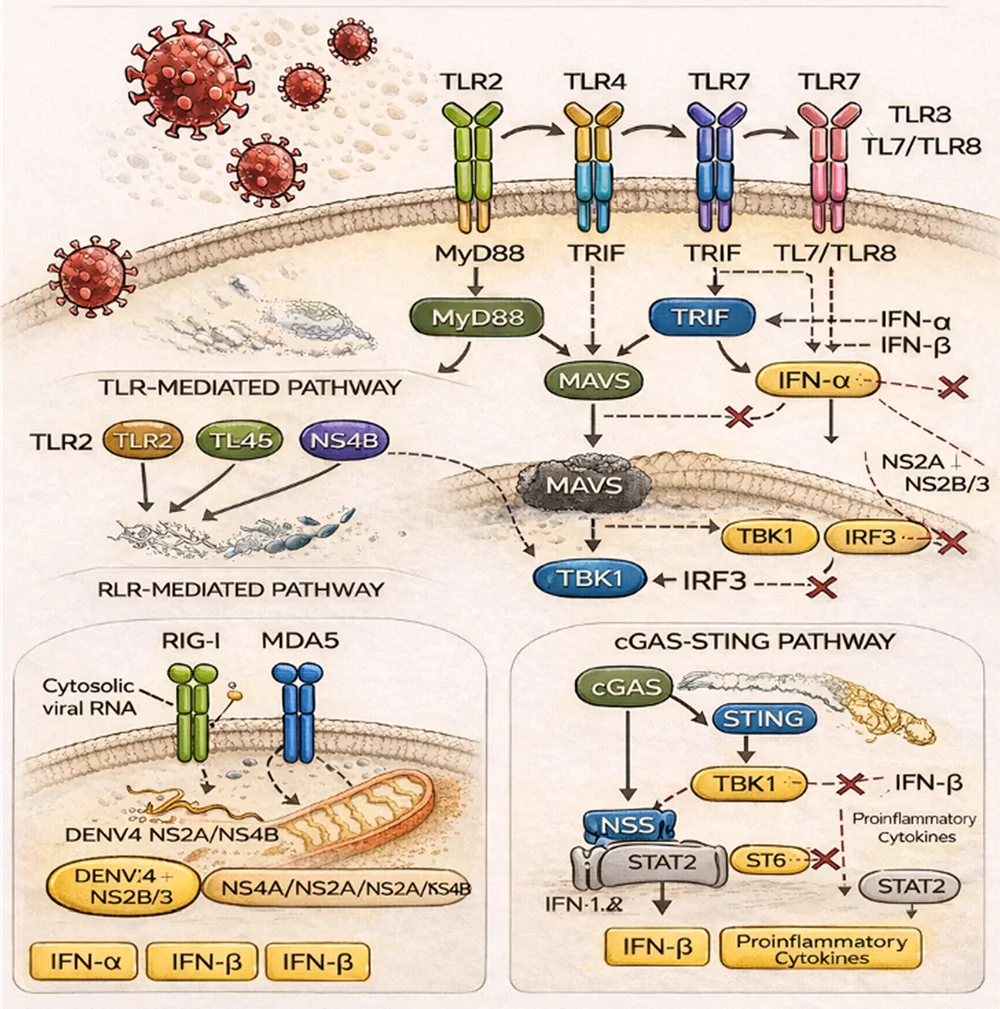
Modulation of innate immune signaling pathways during dengue infection. DENV NS2A/4B restricts TBK1 regulated IFN‐B production, while escape STING‐regulated IFN‐B production. DENV NS2B/3 protease induces the cleavage of STING and DENV NS5 facilitates the cleavage of STAT2, results in the suppression of interferon‐stimulated gene expression.

With regard to RLR signaling, NS3 is able to prevent the translocation of RIG‐I by interacting with the chaperone 14‐3‐3ε, while it has been stated that NS4A interferes with the association between RIG‐I and MAVS [38, 39]. Moreover, NS2A and NS4B have been found to inhibit the activation of TBK1 and the subsequent phosphorylation of IRF3, thus decreasing the production of IFN‐β. NS2A and NS4B from the DENV serotypes 1, 2, and 4 inhibit RIG‐I/MAVS‐associated signaling, whereas NS4A plays a particularly important role in DENV1 [40]. It therefore appears that interference with the RLR–MAVS–TBK1 axis is an important immune‐evasion strategy, even though the extent to which individual viral proteins contribute may differ among serotypes.

DENV also attacks the cGAS–STING pathway at several points. It has been stated that NS2B promotes the degradation of cGAS via an autophagy–lysosomal mechanism, which may reduce the detection of host cytosolic DNA, including mitochondrial DNA. At the same time, the NS2B/3 protease cleaves human STING, thus disrupting the subsequent TBK1–IRF3 signaling and restricting the production of type I interferons [36, 41]. These results indicate that DENV is able to interfere with both the generation of innate immune signals that are DNA‐dependent and with their transmission downstream. Yet, since DENV is an RNA virus, this pathway should be viewed in the context of the host detecting DNA rather than involving direct recognition of DENV RNA by cGAS.

The way in which DENV affects TLR signaling is more complicated. While TLR3 and TLR7 can play a role in the antiviral response to DENV, changes in TLR‐associated inflammatory signaling have also been connected with disease severity [42, 43]. Instead of supposing that DENV uniformly inhibits TRIF‐ or MyD88‐dependent signaling, the available evidence indicates that TLR responses are affected by the viral load, the type of cell, and the overall inflammatory environment. It is important to make this distinction since TLR activation can lead to both the production of antiviral interferons and inflammatory responses.

Downstream of interferon production, DENV interferes with JAK–STAT signaling through several nonstructural proteins. NS2A, NS4A, and NS4B have been implicated in inhibition of STAT1 phosphorylation, whereas NS5 promotes STAT2 binding and proteasomal degradation, thereby impairing formation of the STAT1–STAT2–IRF9 complex and subsequent ISG transcription [44]. Collectively, DENV approaches these modulation strategies for viral evasion since the dengue antigen contributes to impaired immune responses and virus pathogenesis.

## 5 Death Receptor Signaling and Programmed Cell Death During DENV Infection

Death‐receptor signaling plays an important role in regulating immune responses in the case of DENV infection and can help in the elimination of infected cells as well as cause tissue injury. When death receptors such as Fas, TNFR1 and TRAIL‐Rs are engaged, extrinsic apoptosis can be initiated by the recruitment of adapters and by the activation of caspase‐8 (Figure 3). For Fas and TRAIL‐Rs, ligand binding promotes formation of the death‐inducing signaling complex, which contains Fas‐associated death domain protein (FADD) and procaspase‐8 [45]. The activated caspase‐8 then activates the executioner caspases, especially caspase‐3 and caspase‐7, thus causing the typical biochemical and morphological changes associated with apoptosis [46]. Apoptosis may help in the removal of infected cells during DENV infection; yet, excessive or poorly regulated cell death may also result in tissue injury. The net effect is therefore determined by the type of infected cell, the stage of the disease, and the balance between viral clearance and host damage.

**Figure 3:**
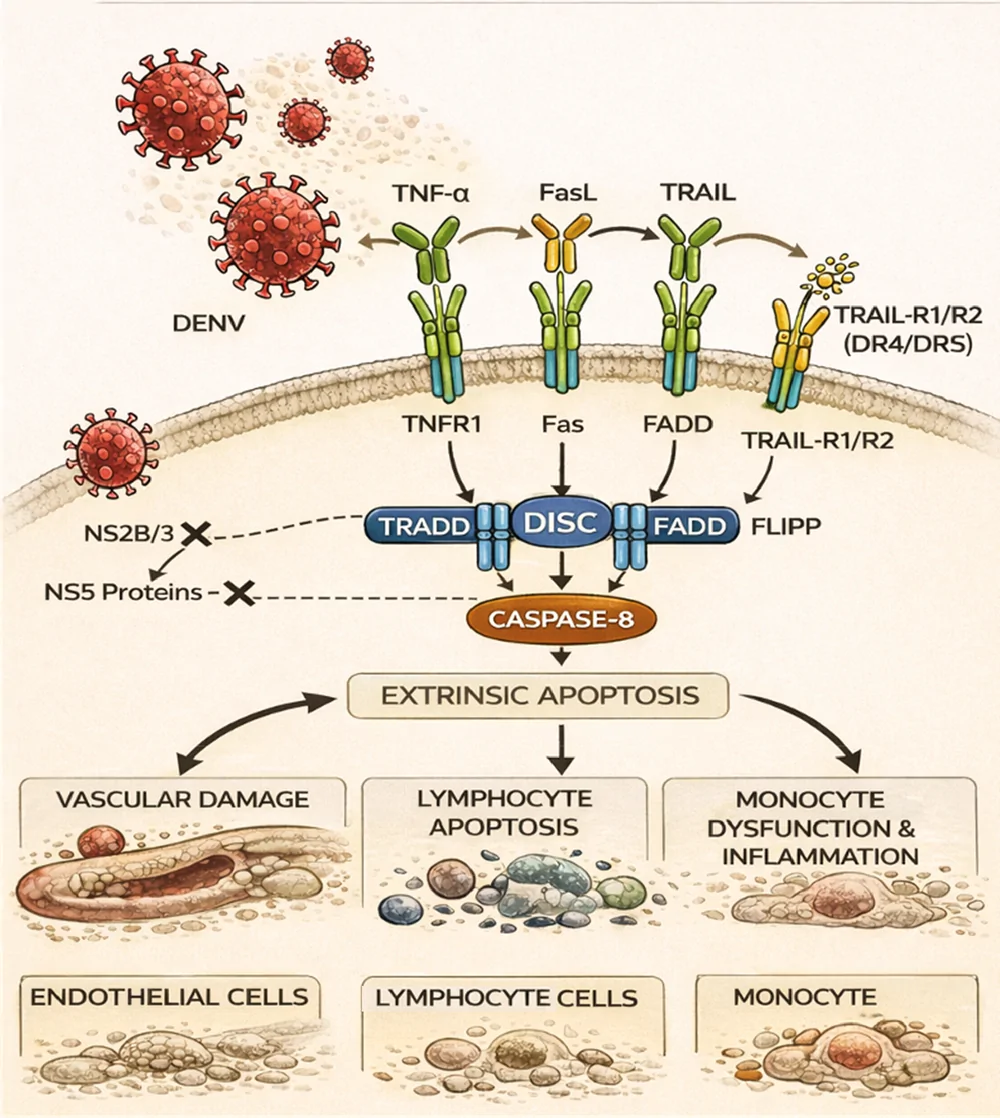
Death receptor pathways in dengue infection. The activation of death receptors causes the execution of apoptosis of diseased cells through the activation of effector caspases, which leads to vascular leakage, lymphocyte apoptosis, and monocyte dysfunction and inflammation. DENV NS2B/3 protease complex and NS5 proteins antagonize apoptosis to trigger viral replication.

It is important to differentiate apoptosis from other types of regulated cell death, especially necroptosis and pyroptosis (Table 3). Apoptosis is a process that depends on caspases and, in its early stages, generally maintains the integrity of the plasma membrane while restricting the release of intracellular contents [47]. On the other hand, necroptosis is a regulated and lytic form of cell death that involves receptor‐interacting protein kinase 3 (RIPK3) and mixed‐lineage kinase domain‐like protein (MLKL), and usually occurs after signaling through receptor‐interacting protein kinase 1 (RIPK1) when the inhibitory action of caspase‐8 is not sufficient [48]. Pyroptosis is an inflammatory form of cell death commonly mediated by inflammatory caspases and gasdermin proteins, which form membrane pores and facilitate the release of intracellular inflammatory mediators [48]. Although these pathways may interact, they are mechanistically different. In the case of DENV infection, their possible roles in causing inflammation and tissue damage should be assessed separately, since the fact that a particular pathway is involved in other viral infections does not mean that it contributes to dengue pathogenesis.

**Table 3:** Cell‐death pathways and potential outcomes during DENV infection.

Cell‐death pathway	Key components	Potential biological outcome
Extrinsic apoptosis	Fas, TNFR1, TRAIL‐Rs, FADD, caspase‐8, caspase‐3/7	Infected‐cell elimination; potentially tissue injury
Intrinsic apoptosis	Mitochondrial signaling, BAX/BAK, caspase‐9	Interference with RIG‐I–MAVS association in specific experimental systems
Necroptosis	RIPK1, RIPK3, MLKL	Inflammatory and lytic cell death
Pyroptosis	Inflammatory caspases, gasdermins	Membrane pore formation and inflammatory mediator release

*Note:* The cell death pathways and their potential biological outcome implicated in DENV recognition are summarized in this table.

Caspase‐8 also has a regulatory role at the point where cell death and inflammatory signaling come together. Together with its ability to initiate extrinsic apoptosis, caspase‐8 can prevent necroptosis by cleaving and inactivating RIPK1 and RIPK3 [46]. If the activity of caspase‐8 is reduced, this inhibitory effect may disappear and necroptosis can then occur via the RIPK1‐RIPK3–MLKL pathway under suitable cellular conditions [49]. The relationship between caspase‐8 and NF‐κB is likewise dependent on the specific context. Signaling through TNFR1 can activate NF‐κB via receptor‐associated signaling complexes, leading to the production of inflammatory mediators and cell‐survival proteins, such as cellular FLICE‐like inhibitory protein and inhibitors of apoptosis proteins [50]. These survival signals can suppress apoptosis, while other arrangements of the signaling complexes may promote cell death. Moreover, the complexes associated with caspase‐8 can also take part in inflammatory signaling, depending on their molecular makeup and the cellular context. Therefore, caspase‐8 is not merely an initiator of apoptosis; its interactions with the signaling proteins it associates with can affect cell survival, inflammatory responses, and the choice of cell‐death pathway. Nevertheless, these general mechanisms should not be taken as proof that each and every interaction has been shown directly in cells infected with DENV.

In the context of DENV infection, the relationship between death‐receptor signaling, antiviral defense, and inflammatory injury is of particular significance. Apoptosis can play a role in the removal of infected cells, and molecules associated with death receptors may also have antiviral effects which do not have to rely on apoptosis. On the other hand, too much cell death or excessive inflammatory signaling might possibly lead to tissue damage. It is not possible to deduce these outcomes simply from measuring death‐receptor activation or caspase‐8 levels. Proof of apoptosis must be based on signs that apoptotic execution has taken place, and assertions regarding necroptosis or pyroptosis need specific evidence for the respective pathways, such as activation of RIPK3–MLKL for necroptosis or activation of inflammatory caspases and cleavage of gasdermin for pyroptosis. Hence, further studies that are specific to DENV are required in order to clarify the relative contributions of these pathways to disease severity and to find out whether selective modulation of them could enhance viral control without at the same time compromising protective immunity.

## 6 Vaccine Development and Therapeutic Strategies Targeting Innate Immune Signaling and Apoptosis in Dengue Virus Infection

The innate immune signaling pathways have a dual function during DENV infection: they contribute to antiviral defense but, if dysregulated or if they become excessive, may also lead to immunopathology. Because of this, they are important targets for the development of vaccines and for therapeutic intervention. Gaining an understanding of how the innate immune signaling pathways interact with cell death receptor‐mediated pathways could yield insights into ways of boosting antiviral immunity while at the same time reducing the inflammation and tissue damage associated with DENV.

### 6.1 Dengue Vaccine Development: Targeting Innate Immune Signaling

Two dengue vaccines, CYD‐TDV (Dengvaxia) and TAK‐003 (Qdenga), have been licensed in different jurisdictions, but their use is influenced by vaccine design, pre‐existing immunity, and serotype‐specific protection (Table 4). Concerning Dengvaxia, a significant safety issue has been identified in that people who have no prior history of DENV infection may be at a higher risk of developing severe dengue should they then have a natural infection, which is why it is necessary to have reliable evidence of a previous infection before administering the vaccine in accordance with the relevant guidelines. TAK‐003 is a live‐attenuated tetravalent vaccine based on a DENV‐2 backbone and is recommended by the World Health Organization (WHO) for children between 6 and 16 years of age in areas where dengue transmission is intense. Nevertheless, vaccine efficacy varies between serotypes and depends on the baseline serostatus, and protection against all four serotypes has not been uniformly established [51, 52].

**Table 4:** Comparison of licensed dengue vaccines and key safety considerations.

Feature	Dengvaxia (CYD‐TDV)	Qdenga (TAK‐003)
Platform	Live‐attenuated chimeric vaccine using a yellow fever 17D backbone	Live‐attenuated tetravalent vaccine using a DENV‐2 backbone
Dosing	Three doses at 0, 6, and 12 months	Two doses, 3 months apart
Serostatus	Requires evidence of previous DENV infection under relevant recommendations	WHO recommends use in children aged 6–16 years in settings with high dengue transmission intensity
Efficacy consideration	Vaccination of seronegative individuals can increase the risk of subsequent severe dengue; prevaccination screening is important	Protection varies by serotype and baseline serostatus; protection against some serotypes in seronegative recipients remains a consideration
Current status	Discontinued by the manufacturer due to low global demand; no longer available in the United States	WHO‐recommended for specified populations and settings; national authorizations and recommendations vary
Key safety issue	Risk of vaccine‐associated enhanced disease in recipients without prior infection	Continued monitoring for hypersensitivity, anaphylaxis, and potential vaccine‐associated enhanced disease

*Note:* The comparison between licensed dengue vaccines based on different features is summarized in this table.

The activation of the innate immune system is a key part of the immunity produced by vaccines. Adjuvants which trigger certain innate immune pathways can help to activate dendritic cells, promote antigen presentation, and thus lead to T‐cell and B‐cell responses [53]. Yet the effects of adjuvants depend on their molecular targets, the dose used, the formulation, and the vaccine platform. Even though TLR agonists are being studied as immunostimulatory adjuvants in the development of vaccines, their possible use in dengue vaccines needs a specific assessment of immunogenicity and safety [54]. In particular, greater immune activation should result in protective responses without causing excessive inflammation or damaging the quality of the antibody responses.

The development of future dengue vaccines should therefore focus on achieving balanced, long‐lasting, and serotype‐inclusive protection, taking into account pre‐existing immunity and the possible risk of antibody‐dependent enhancement [55]. Antibody quantity alone is insufficient to establish protective efficacy; the specificity, functional activity, and durability of antibody responses are also important. By combining these factors with an investigation of innate immune signatures, it may be possible to assist in the design and assessment of next‐generation dengue vaccines. However, candidate adjuvants and other strategies that target innate immune pathways should be differentiated from those approaches which have already been validated in licensed dengue vaccines.

### 6.2 Therapeutic Strategies: Targeting of Innate Immune Signaling

Therapeutic strategies targeting innate signaling pathways focus on reversing protective defense mechanisms mediated by DENV or restricting hyperinflammation (Table 5). Experimental approaches have been studied using STING agonists such as cAIMP and diABZI or TLR agonists because the activation of innate immune sensors can lead to type I IFN responses and thus limit flavivirus replication [56, 57]. Just as exogenous IFN‐α or IFN‐β has shown antiviral activity against DENV in experimental models, the therapeutic value of interferon‐based methods will depend on the time of treatment, the dose used, the cellular context, and the possibility of causing excessive inflammatory responses [58]. The DENV NS2B/3 protease is involved not only in the processing of the viral polyprotein but also in the cleavage of human STING, which makes NS2B/3 a highly promising target for the development of antiviral drugs. It therefore follows that inhibiting this protease would be able to suppress viral replication while at the same time preserving STING‐mediated innate immune signaling [9]. NS2B/3 inhibitors could therefore potentially suppress viral replication while limiting this particular mechanism of immune evasion. Nevertheless, their therapeutic value depends on achieving selective inhibition, adequate intracellular activity, and acceptable safety; further preclinical and clinical evaluation is required for establishing clinical efficacy.

**Table 5:** Therapeutic strategies targeting DENV replication, innate immune signaling, and cell‐death pathways: proposed mechanisms, evidence status, and limitations.

Therapeutic strategy		Proposed mechanism	Evidence status	Safety concerns
NS2B/NS3 protease inhibitors		Inhibit viral polyprotein processing and suppress viral replication.	Predominantly biochemical, cell‐based, and preclinical research; no approved DENV protease inhibitor.	Selectivity, intracellular potency, pharmacokinetics, toxicity, and efficacy after symptom onset.
STING agonists		Activate STING–TBK1–IRF3 signaling and promote type I interferon responses.	Experimental for DENV‐directed therapy; clinical efficacy in dengue has not been established.	Excessive inflammation, systemic toxicity, and cell‐type‐dependent responses.
TLR agonists		Stimulate innate immune signaling and potentially enhance antiviral responses and antigen presentation.	Investigational as DENV‐directed therapeutics; some TLR agonists have been evaluated in other clinical settings.	Cytokine release, inflammatory injury, and context‐dependent effects on adaptive immunity.
Interferon‐based therapy		Enhance interferon‐stimulated gene expression and establish an intracellular antiviral state.	Antiviral activity investigated in experimental systems; no established routine interferon treatment for dengue.	Treatment timing, systemic adverse effects, and uncertain benefit during established inflammatory disease.
Apoptosis‐modulating agents		Alter caspase activation, BCL‐2 family signaling, or death‐receptor pathways.	Experimental for DENV; clinical benefit has not been established.	Unintended tissue injury, impaired removal of infected cells, and disruption of immune‐cell or endothelial‐cell survival.
SMAC mimetics/IAP inhibitors		Modulate IAP‐associated signaling and caspase activity, potentially promoting cell death or altering inflammatory responses.	Investigated in other disease settings; application to DENV remains experimental.	Uncontrolled cell death, inflammatory signaling, tissue injury, and uncertain effects on viral clearance.
TRAIL‐receptor agonists		Activate DR4/DR5‐mediated extrinsic apoptosis.	Experimental candidate strategy; clinical efficacy against dengue has not been established.	Potential injury to uninfected cells, uncertain cell‐type selectivity, and possible effects on endothelial integrity.

*Note:* The proposed mechanisms, evidence status, and safety concerns of different therapeutic strategies are summarized in this table.

On the other hand, selective modulation of excessive inflammatory signaling—such as that associated with TNF‐α and NF‐κB—might offer a possible host‐directed strategy for reducing immunopathology and vascular dysfunction. Yet, as a general suppression of these pathways might impair antiviral immunity, affect the survival of immune cells, or delay the clearance of the virus, similarly, approaches that modulate apoptosis—such as those which target members of the BCL‐2 family, inhibitor of apoptosis proteins (IAPs), caspases, or TRAIL receptors—could have an effect on the elimination of infected cells. On the other hand, improper manipulation of the cell‐death pathways might cause damage to uninfected tissues, lead to inflammatory cell death, or enable infected cells to persist. Accordingly, their use in dengue is still experimental and needs to be assessed in models that are relevant to the disease.

The therapeutic window for these methods is probably going to vary according to the stage of the disease, the dose used, the degree of selectivity for the pathway, the distribution among tissues, and the balance between viral control and damage caused by the host's immune response. The early boosting of antiviral responses will probably have different effects from immune modulation in the case of an already established inflammatory disease. Future research therefore needs to evaluate not only the antiviral effect but also the inflammatory toxicity, the injury to the endothelium, the function of immune cells, and clinically important outcomes. Until an intervention has shown sufficient efficacy and safety in clinical trials, supportive care will continue to be the standard of treatment for dengue.

### 6.3 Apoptosis‐Related Interventions

Apoptosis, or programmed cell death, is a sophisticated cellular process for maintaining tissue homeostasis and dying diseased or infected cells. During DENV, apoptosis demonstrates multifaceted roles: it can restrict viral amplification by killing infected cells, but uncontrolled cell death can lead to excessive tissue damage or injury, which results in vascular damage and severe disease outcomes [37]. Therapeutic strategies aiming at programmed cell death can create a fine‐tuned immune response by balancing tissue damage and the antiviral defense state, either by suppressing the rate of cell death or stimulating the death of diseased cells, mainly depending on the disease context [59]. When excessive cell death contributes to DENV immunopathology, including endothelial cell damage or tissue injury, inhibitors of the pan‐caspase family, for example z‐VAD‐FMK, can be used in experiments to suppress caspase‐dependent apoptosis and have therefore been helpful in determining the role of apoptosis in cellular injury associated with DENV. Yet their therapeutic value in dengue is still purely hypothetical since systemic inhibition of apoptosis might interfere with the elimination of infected cells and other host‐protective functions [60]. Furthermore, the antiapoptotic gene Bcl‐2 appears to stimulate the stabilization of the mitochondrial membrane, limiting the premature cell death.

On the other hand, it is theoretically possible to promote the elimination of infected cells by using drugs to enhance apoptosis; a practical example of this is given by inhibitors of the BCL‐2 family such as venetoclax; yet their use in the case of dengue is still experimental and demands careful assessment since the uncontrolled induction of apoptosis could worsen tissue injury [61]. It is established that IAPs inhibit caspases. Xevinapant is able to influence IAP‐related signaling, and agonists of the TRAIL receptors DR4 and DR5 can trigger the extrinsic apoptotic pathways. However, these compounds have not yet demonstrated clinical efficacy in the case of dengue, and their effects on both infected and uninfected cells must be carefully evaluated [62].

Promising therapeutic strategies for DENV significantly focus on host‐directed therapies, which particularly modulate host signaling pathways such as apoptosis, interferon‐stimulated gene response, and inflammatory cytokine signaling rather than defending the virus specifically [63]. The therapeutic window must be taken into account since an inhibition of apoptosis could enable infected cells to continue to exist, while unselective activation of cell death might exacerbate tissue injury or impair the survival of immune and endothelial cells. It also cannot be assumed that TRAIL‐receptor agonists will selectively destroy infected cells without having an effect on uninfected tissues as well. Future research therefore needs to determine the cell‐type specificity, the dose‐dependent effects, the timing, the antiviral efficacy, and the safety of apoptosis‐modulating interventions in cellular and animal models relevant to DENV. The aim is to maintain the protective antiviral responses while at the same time restricting pathological cell death, not merely increasing or suppressing apoptosis.

### 6.4 Translational Perspective

Taken together, the innate immune and cell death pathways described in this review offer a number of possible targets for the development of dengue vaccines and antivirals. Stimulating RLRs, TLRs, and cGAS–STING signaling pathways can lead to the production of type I interferons, promote antigen presentation, and thus trigger adaptive immune responses, indicating that a controlled activation of these pathways could be of value in the design of rational vaccines and adjuvants [64]. On the other hand, over‐activation of inflammatory signaling might contribute to immunopathology, underscoring the need to achieve a proper balance between antiviral immunity and inflammation. In contrast, the DENV proteins which antagonize the innate immune response are promising targets for antiviral development [65]. Specifically, the disruption of STING signaling by NS2B/3, the interference with upstream antiviral signaling by NS4A/NS4B, and the antagonism of JAK–STAT signaling by NS5 all provide a mechanism by which to restore the host's antiviral responses. More recent studies stress that these viral immune‐evasion mechanisms could be used to guide the creation of both direct‐acting antivirals and the next‐generation live‐attenuated tetravalent vaccines [66].

The fact that cell death pathways offer a further avenue for host‐directed therapy means that instead of generally inhibiting apoptosis, future methods should seek to selectively regulate death receptor and caspase signaling depending on the stage of the disease and the cellular context. This could aid in keeping the clearance of infected cells by antiviral mechanisms while at the same time reducing excessive tissue damage and inflammatory reactions [67].

Hence, it is necessary to determine the therapeutic window, dosage, timing, and their possible effects on viral clearance before these methods can be taken into consideration for use in a clinical setting. Currently, methods that aim at innate immune signaling, viral immune‐evasion mechanisms, and apoptosis are still under investigation for dengue, and none of them has proved good enough in a clinical setting to take the place of current supportive care. In the end, a reasonable basis for future combination therapies could be achieved by combining direct inhibition of viral replication with a carefully chosen modulation of the host's response. Yet, successful translation of these approaches will rely on showing that they reduce the severity of the disease without impairing viral clearance or leading to further tissue damage.

## 7 Research Gap and Future Directions

Despite sophisticated advancements in understanding the mechanism of DENV pathogenesis, there are critical research gaps and unanswered questions still remained. Although it has been studied that the key nonstructural proteins such as NS2B/3, NS4A/B and NS5 are involved in immune evasion while allowing the viral spread and disease clinical outcomes. A deeper research on how the viral proteins differentially modulate the innate immune signaling, such as TLR, RLR and cGAS‐STING pathways across numerous immune and endothelial vascular cells is crucial. A research gap remains in the precise mechanism explaining the dysregulation of innate immune signaling and cell death receptor pathways to virus replication and disease severity.

Another critical gap remains in differentiating antiviral defense versus immunopathology. Type I interferon responses are indispensable for the protective response, but they are associated with inflammation and endothelial cell damage. Similarly, cell death receptor‐induced apoptosis can limit viral spread, but enhance immune and endothelial cell damage and vascular leakage. The molecular mechanism behind apoptosis determines whether it acts as a defensive or pathogenic immune function during severe infection is not clear.

Furthermore, research is based on in vivo or in vitro systems, which might not clearly reflect the human disease severity. It is essential to develop translational models, such as organoid systems and improved in vivo model to better understand and recapitulate human immune cellular responses. Also, a few biomarkers are available for the disease severity prediction, emphasizing the necessity of stronger multi‐omics approaches to immune profiling, linking gene expression with functional protein and immune response.

Further research should aim at detecting the interrelationship between innate immune pathways and apoptosis, as well as exploring the significance of mitochondrial dynamics and metabolism, and may explore more promising approaches. Understanding these research gaps will be paramount for the development of efficient and safe vaccines and therapeutic strategies targeted at turning down DENV‐associated morbidity and mortality.

## 8 Conclusion

DENV pathogenesis is featured by a dynamic relationship between virus replication and host‐derived innate immune response. Innate immune signals such as RLRs, TLRs, cGAS–STING axis have a significant role in the recognition of viral RNA and the induction of antiviral response through type I interferon and inflammatory immune responses. However, DENV proteins, including NS2B/3, NS4A/4B and NS5, mediate immune modulation through the blockade or inhibition of STING, MAVS, TBK1, IRF3 and STAT2, thereby permitting virus persistence and replication. Additionally, TNFR1, Fas and TRAILR‐mediated cellular death can restrict virus spread, followed by the elimination of infected cells, even though uncontrolled apoptosis is associated with immune cell damage and vascular dysfunction, which is demonstrated as a sign of severe dengue. The bridge between inflammatory signaling and apoptosis underlies the fine‐tuned immune balance between antiviral defense and immunopathology. A deeper knowledge of these connecting pathways is necessary for safer drug development and improving targeted therapies focused on reducing disease severity and worldwide health burden.

## Author Contributions

**Farjana Islam:** conceptualization, writing – original draft, review and editing. The author has fully read and approved the final version of the manuscript.

## Funding

The author has nothing to report.

## Conflicts of Interest

The author declares no conflicts of interest.

## Data Availability

The author has nothing to report.
